# Use of low-dose β_1_-blocker for sinus tachycardia in patients with catecholamine support following cardiovascular surgery: a retrospective study

**DOI:** 10.1186/s13019-019-0966-z

**Published:** 2019-07-25

**Authors:** Michihiro Sakai, Satoshi Jujo, Junjiro Kobayashi, Yoshihiko Ohnishi, Masataka Kamei

**Affiliations:** 1 Department of Clinical Anesthesiology, Mie University Hospital, 2-174 Edobashi, Tsu, Mie 514-8507 Japan; 2 Department of Anesthesiology, National Cerebral and Cardiovascular Center, 5-7-1 Fujishiro-dai, Suita, Osaka, 565-8565 Japan; 3 Department of Cardiovascular Surgery, National Cerebral and Cardiovascular Center, 5-7-1 Fujishiro-dai, Suita, Osaka, 565-8565 Japan

**Keywords:** Tachycardia, Catecholamine, Cardiovascular surgery

## Abstract

**Background:**

Sinus tachycardia coupled with high-dose catecholamine is common after cardiopulmonary bypass (CPB). The present study assessed the hemodynamic efficacy and safety of combination therapy using low-dose β_1_-selective adrenergic blocker (landiolol) and inotropes.

**Methods:**

This was a retrospective, single center, self-comparison study at post-anesthesia care unit within a tertiary care center. The study included adults who underwent cardiac surgery with CPB and received landiolol between April 2007 and November 2011. We assessed hemodynamic data prior to and 1 h after initiation of landiolol therapy.

**Results:**

We evaluated 11 patients who were administered 2.6 ± 1.3 μg/kg/min (mean ± SD) landiolol with sinus tachycardia and received catecholamine therapy after on-pump cardiovascular surgery. Landiolol administration led to a significant reduction in heart rate (HR; 112.4 ± 5.8 vs 126.0 ± 7.6 beats/min, *p* < 0.001), and a significant increase in stroke volume index (SVI) assessed by pulmonary artery catheterization (22.4 ± 5.4 vs. 18.9 ± 4.2 mL/m^2^, *p* = 0.04). Only one patient showed no HR reduction, whereas seven patients showed decreased HR and increased SVI (64, 95% confidence interval: 30–98%). Moreover, all five patients who received high-dose catecholamine support showed improved hemodynamics. In terms of safety, no patients required cessation of landiolol therapy.

**Conclusions:**

Low-dose landiolol therapy may safely decrease HR and improve hemodynamics among patients with sinus tachycardia receiving catecholamine treatment after cardiovascular surgery.

**Trial registration:**

This study is retrospective. Registration number: 11. Duration of registration: April 2007~November 2011.

## Background

Sinus tachycardia coupled with high-dose catecholamine is a common problem after cardiopulmonary bypass (CPB). Since tachycardia is associated with mortality and cardiac morbidity in the general population [1, 2] as well as in patients following surgery [3, 4], heart failure [5], and/or coronary artery disease [6], anesthesiologists should carefully manage heart rate (HR) throughout the perioperative period.

Catecholamine therapy after CPB aids cardiac performance by stabilizing hemodynamic conditions. However, high-dose catecholamine therapy frequently induces sinus tachycardia, and its treatment remains highly controversial. Continuation of tachycardia may compromise the myocardial oxygen balance, ultimately followed by global cardiac performance deterioration. However, reducing inotropic stimulation can induce a decrease in cardiac output, followed by deterioration of hemodynamic conditions. Therefore, an effective approach that avoids this vicious circle is necessary.

Landiolol is an injectable, ultra-short-acting, highly selective β_1_-adrenoreceptor blocker with a potency ratio (β_1_/β_2_) of 255 and an elimination half-life value of 4 min in healthy individuals, compared with esmolol, which has a potency ratio of 33 and elimination half-life of 9 min [7, 8]. Since landiolol is more likely to have high β_1_-selectivity and a short half-life compared with esmolol or other β_1_-blockers, a bolus shot of landiolol may be used to safely attenuate tachycardia in response to endotracheal intubation under general anesthesia [9, 10]. Recent studies have demonstrated the efficacy and safety of continuous intravenous landiolol infusion in different clinical settings to treat atrial fibrillation and/or atrial flutter after cardiac surgery [11–13] as well as acute heart failure [14]. However, these reports excluded unstable hemodynamic cases requiring high-dose catecholamine support. There have been some reports of clinical observations of combination therapy with β-blocker and low-dose inotrope in decompensated heart failure [15]. However, to date, the hemodynamic efficacy and safety of ultra-short-acting β_1_-blockers remains largely unclear in cases with concomitant administration of high-dose inotropes. We hypothesized that ultra-short-acting β_1_-blocker therapy could represent a valuable therapeutic option in cases of sinus tachycardia under high-dose catecholamine support. As a preliminary investigation, we conducted a retrospective, single center, self-comparison study.

## Methods

### Research design

The Research Ethics Review Board of National Cerebral and Cardiovascular Center approved the present study. Due to the use of de-identified data, informed consent was waived. Cardiac surgical intensive care unit charts for all adult patients who underwent on-pump cardiovascular surgery between April 2007 and November 2011 at the National Cerebral and Cardiovascular Center Hospital, Suita, Osaka were reviewed. The inclusion criteria were as follows: (1) patients who had persistent sinus tachycardia (HR > 100 beats/min) induced by catecholamine support after CPB; (2) patients who received continuous intravenous landiolol for sinus tachycardia based on the decision of the attending physician; (3) landiolol administration overlapped with constant dose of inotropic drugs (catecholamine, vasodilator, and/or phosphodiesterase 3 inhibitor) for at least for 1 h; (4) patients who had a pulmonary artery catheter; and (5) patients who were sedated and supported via mechanical ventilation. The exclusion criteria were as follows: (1) patients who had a temporary or permanent pacemaker; (2) patents who had any atrial arrhythmia except for sinus tachycardia; or (3) patients who had any mechanical circulatory support, including continuous renal replacement therapy.

### Assessment of clinical features

In addition to general clinical features, we collected data on preoperative EuroSCORE, preoperative left ventricular ejection fraction, and fractional shortening via transthoracic echocardiography.

To comprehensively assess the various regimens of catecholamine, we used a modified Wernovsky inotrope score [16], calculated using the following formula: cardiac agent index (CAI) = dopamine dose (μg/kg/min) + dobutamine dose (μg/kg/min) + 100 × epinephrine dose (μg/kg/min) + 100 × norepinephrine dose (μg/kg/min) + 10 × phosphodiesterase 3 inhibitor dose (μg/kg/min). High-dose catecholamine use was defined as CAI > 10. Definitions of hemodynamic instability and the approaches used to maintain hemodynamic conditions were based on the assessment by individual anesthesiologists.

To evaluate adverse events, plasma creatine kinase-MB isozyme levels were measured every 6 h, as part of the routine daily clinical practice at our intensive care unit.

### Hemodynamic parameters

We evaluated hemodynamic variables just before and 1 h after landiolol infusion. Using a 7.5 Fr. Continuous Cardiac Output Thermodilution Catheter (Edwards Lifescience, CA, USA) and TruWave Disposable Pressure Transducer (Edwards Lifescience, CA, USA), the following variables were measured: HR (beats/min), cardiac output (L/min), cardiac index (CI, L/min/m^2^), stroke volume (SV, mL), stroke volume index (SVI, mL/m^2^), mixed venous oxygen saturation (SvO_2_, %), systolic arterial blood pressure (mmHg), diastolic arterial pressure (mmHg), mean arterial blood pressure (mmHg), systolic pulmonary artery pressure (mmHg), diastolic pulmonary artery pressure (mmHg), mean pulmonary artery pressure (mmHg), and central venous pressure (mmHg). Blood temperature (BT, °C) was also measured.

### Statistical analysis

All data are presented as mean ± SD unless otherwise stated. Paired *t* test was used to compare hemodynamic variables. Pearson’s correlation coefficient (r) was used to study the relationship between changes in rate of HR reduction and SVI elevation. All *p*-values < 0.05 (two-tailed) were considered significant. Statistical analyses were performed using SPSS software version 11.0.1 J (IBM SPSS Inc., IL, USA).

## Results

### Patient characteristics

Table 1 summarizes the clinical characteristics of the patients.

**Table 1:** Baseline characteristics of the patients

Patients	1	2	3	4	5	6	7	8	9	10	11	Mean ± SD
Age (years)	73	25	39	23	76	62	34	68	78	68	28	52.2 ± 22.2
Gender (M/F)	M	F	M	F	F	F	M	M	M	F	M	
Height (cm)	153	166	170	167	155	161	182	162	150	150	177	163.0 ± 10.7
Weight (kg)	47	48	95	55	56	62	72	52	60	38	85	60.9 ± 17.0
LVEF (%)	72	63	63	63	58	48	63	55		81	33	59.9 ± 13.0
FS (%)	43	40	40	40	28	33	37	29		53	14	35.7 ± 10.5
Emergency (Yes/No)	No	No	Yes	No	Yes	No	Yes	No	Yes	No	No	
CPB time (min)	191	356	351	289	178	240	369	211	180	246	138	250 ± 81
EuroSCORE	7	8	5	7	10	6	7	4	11	6	3	6.7 ± 2.4
CAI	6.6	8.4	11.8	4.6	3.1	41	22.1	5.4	18.2	36.4	3	14.6 ± 13.4
Landiolol (μg/kg/min)	1.8	1.6	1.5	2.3	2.3	2.1	2.0	4.4	4.2	1.1	5.0	2.6 ± 1.3

CAI was calculated using the following formula: CAI = dopamine dose (μg/kg/min) + dobutamine dose (μg/kg/min) + 100 × epinephrine dose (μg/kg/min) + 100 × norepinephrine dose (μg/kg/min) + 10 × phosphodiesterase 3 inhibitor dose (μg/kg/min). High-dose catecholamine use was defined as use of CAI > 10. No preoperative LVEF and FS values were measured in patient no. 9 due to emergency operation, but global systolic function was good assessed by transesophageal echocardiography intraoperatively

*F* female, *M* male, *LVEF* left ventricular ejection fraction, *FS* fractional shortening, *CPB* cardiopulmonary bypass, *CAI* cardiac agent index

We identified 11 out of 1762 adult patients who underwent on-pump cardiovascular surgery (0.6%). The average continuous infusion dose of β_1_-blocker landiolol was 2.6 ± 1.3 μg/kg/min (range, 1.1–5.0 μg/kg/min), indicating that our dose was lower than the manufacturer’s recommended dose for tachycardia prevention (20–40 μg/kg/min). All landiolol infusions were started without an initial loading dose. No bolus shots or up- or down-titrations were used, even beyond the observation phase. The average CAI value was 14.6 ± 13.4. Five of the 11 patients (45%) received high-dose catecholamine therapy (> 10), but none of these used epinephrine.

### Hemodynamic evaluation

Landiolol administration led to a reduction in HR by approximately 11%, and was significantly different (Table 2) within our study population (*p* < 0.001). Notably, administration of low-dose landiolol with concomitant used of inotropes led to an 18.5% increase in SVI (*p* = 0.04), with no deterioration in hemodynamic parameters.

**Table 2:** Hemodynamic parameters of the 11 patients

	Pre-landiolol	Post-landiolol	*p*-value
HR	126.0 ± 7.6	112.4 ± 5.8	< 0.001
SVI	18.9 ± 4.2	22.4 ± 5.4	0.04
SV	30.8 ± 6.6	36.7 ± 10.5	0.033
CI	2.4 ± 0.5	2.5 ± 0.6	0.32
CO	3.9 ± 0.8	4.1 ± 1.1	0.28
SvO_2_	63.1 ± 12.8	65.2 ± 13.3	0.42
sBP	86.6 ± 29.1	102.8 ± 19.6	0.082
dBP	54.9 ± 10.5	59.3 ± 8.7	0.11
mBP	80.3 ± 18.8	81.5 ± 15.7	0.71
sPAP	28.6 ± 5.1	29.6 ± 8.8	0.51
dPAP	20.3 ± 4.9	18.9 ± 5.6	0.14
mPAP	21.1 ± 7.6	22.5 ± 6.5	0.53
CVP	10.4 ± 3.6	10.5 ± 5.1	0.91
BT	37.4 ± 0.8	37.6 ± 0.6	0.17

Values represent mean ± SD. Change in HR is one of the coprimary end points

*HR* heart rate (beats/min), *SVI* stroke volume index (mL/m^2^), *SV* stroke volume (mL); *CI* cardiac index (L/min/m^2^), *CO* cardiac output (L/min), *SvO*_*2*_ mixed venous oxygen saturation (%), *sBP* systolic arterial blood pressure (mmHg), *dBP* diastolic arterial pressure (mmHg), *mBP* mean arterial blood pressure (mmHg), *sPAP* systolic pulmonary artery pressure (mmHg), *dPAP* diastolic pulmonary artery pressure (mmHg), *mPAP* mean pulmonary artery pressure (mmHg), *CVP* central venous pressure (mmHg), *BT* blood temperature (°C)

**Fig. 1:**
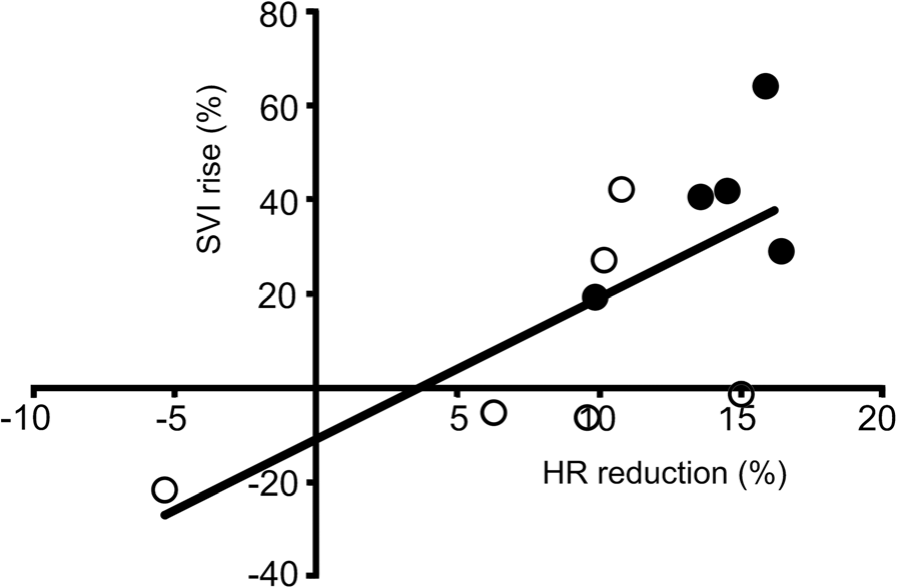
Relationship between change rates in heart rate reductions and stroke volume index increases for 11 patients. The following formula was used to calculate change rates: Change rate = (post-landiolol value – pre-landiolol value) / pre-landiolol value × 100 (%). Heart rate reduction showed a negative change rate. Closed circles indicate data from participants who received high-dose catecholamine support. The study treatment was considered clinically acceptable in terms of hemodynamic efficacy (one of the coprimary end points) (r = 0.7, *p* = 0.016). HR reduction, negative change rate of heart rate; SVI rise, change rate of stroke volume index

A correlation between HR and SVI was observed (Fig. 1), and there was no evidence that SVI deterioration was caused by HR reduction. HR increased in response to landiolol in only one case (patient no. 1), followed by a decrease in SVI, suggesting that excess tachycardia harmed output. Three cases showed no changes in SVI despite reductions in HR (patient nos. 2, 5, and 8).

Plasma creatine kinase-MB isozyme levels did not differ significantly between pre- and post-landiolol administration (142.3 ± 271.5 vs. 199.9 ± 424.6 ng/mL, respectively; *p* = 0.31).

### Subgroup analysis

Five patients received high-dose catecholamine support (CAI > 10), and are shown as a subgroup in Table 3. HR reduction led to an increase in SVI as well as improved CI, suggesting that low-dose landiolol may not only confer control rate ability, but may also exert a positive impact on cardiac function in conjunction with high doses of catecholamine.

**Table 3:** Hemodynamic parameters in the five patients with high-dose catecholamine support

	Pre-landiolol	Post-landiolol	*p*-value
HR	130.4 ± 6.4	112.0 ± 4.5	< 0.001
SVI	17.6 ± 4.6	24.2 ± 5.9	0.007
SV	29.4 ± 8.3	40.3 ± 11.2	0.008
CI	2.3 ± 0.6	2.7 ± 0.6	0.025
CO	3.8 ± 1.0	4.5 ± 1.2	0.029
SvO_2_	57.8 ± 18.0	61.8 ± 19.2	0.50
sBP	85.2 ± 7.9	103.6 ± 20.8	0.75
dBP	48.8 ± 5.8	58.8 ± 7.2	0.012
mBP	84.1 ± 22.4	90.7 ± 14.3	0.22
sPAP	30.2 ± 7.2	33.2 ± 12.7	0.36
dPAP	20.8 ± 7.2	20.0 ± 8.5	0.65
mPAP	23.9 ± 6.7	24.4 ± 9.6	0.83
CVP	11.8 ± 5.0	11.6 ± 7.2	0.91
BT	37.3 ± 0.7	37.5 ± 0.5	0.42

Values represent mean ± SD

*HR* heart rate (beats/min), *SVI* stroke volume index (mL/m^2^), *SV* stroke volume (ml), *CI* cardiac index (L/min/m^2^), *CO* cardiac output (l/min), *SvO*_*2*_ mixed venous oxygen saturation (%), *sBP* systolic arterial blood pressure (mmHg), *dBP* diastolic arterial pressure (mmHg), *mBP* mean arterial blood pressure (mmHg), *sPAP* systolic pulmonary artery pressure (mmHg), *dPAP* diastolic pulmonary artery pressure (mmHg), *mPAP* mean pulmonary artery pressure (mmHg), *CVP* central venous pressure (mmHg), *BT* blood temperature (°C)

## Discussion

The findings of the present study highlight the usefulness of a low-dose infusion of the β_1_-blocker, landiolol, during the acute postoperative phase in patients with sinus tachycardia receiving high-dose catecholamine support. The successful use of landiolol for HR control was shown previously in clinical settings, including atrial fibrillation [17] and sepsis [18]. However, there is some uncertainty regarding administration of β-blockers in patients who require a potent inotropic agent to guard against serious hemodynamic instability [19]. Some reports have shown beneficial effects of intravenous β-blocker in a clinical setting of low-dose catecholamine support [20, 21]. However, to the best our knowledge, no previous trials have examined this issue from the standpoint of high-dose inotropes. In the cases we studied, relatively low-dose landiolol reduced HR and increased SVI without adversely affecting patients treated with high-dose catecholamines.

Catecholamines are the first-line drug treatment to support cardiac performance after CPB. Since excess sinus tachycardia induced by high-dose catecholamine can reduce the duration of coronary perfusion [22] and coronary vasoconstriction [23], efforts must be made to prevent sinus tachycardia following cardiac surgery. Epinephrine is a choice catecholamine used to avoid tachycardia [24], but is not a first-line drug for low cardiac output syndrome treatment due to its detrimental effects on splanchnic blood flow and its ability to induce lactic acidosis [25]. The present study indicates the possibility of using the ultra-short-acting β1-blocker, landiolol, to optimize HR without inhibiting the favorable hemodynamic response brought about by the conventional use of inotropes.

Administration of landiolol should be initiated at a loading dose of 40 μg/kg/min, and then titrated to a maintenance dose. However, this method has been shown to cause hypotension [26]. Additionally, several reports have shown that landiolol administration without an initial loading dose was effective in preventing atrial fibrillation/atrial flutter/paroxysmal atrial tachycardia (maintenance dose range, 1–5 μg/kg/min) in surgical patients with left ventricular dysfunction [27, 28]. Therefore, we did not administer a loading dose to patients receiving high-dose catecholamine support.

Surprisingly, our results suggest that landiolol has multiple beneficial effects, both in terms of controlling HR and positively affecting SVI under high-dose catecholamine support. This is similar to the manner by which a low-dose β-blocker infusion may decrease the undesirable response associated with catecholamine without significantly reversing the desired inotropic effects. Additionally, a slower HR facilitates a lengthier diastolic filling time and subsequently increases the static arterial elastance. Improved ventricular–arterial coupling is thought to increase the SVI [29]. Therefore, accurate evaluation of diastolic function may have a positive impact on HR reduction.

Human ventricular myocardium contains a mixed population of β_1_ and β_2_ adrenergic receptors, and patients with heart failure have lower a β_1_/β_2_ ratio compared with healthy hearts [30]. Moreover, both β_1_ and β_2_ receptors in the ventricle were coupled to a positive inotropic response. Therefore, one can speculate that the β_2_ receptor acts as an inotropic mediator to maintain systolic function in cases where there is concomitant use of landiolol and inotropes. The reaction stemming from combination of landiolol and inotropes may be altered depending upon the population of β-adrenergic receptors in the ventricle. However, landiolol cannot be administered based solely on the molecular evidence regarding the β_1_/β_2_ ratio in the human ventricle. Therefore, careful titration and monitoring of the patient is required to assess the efficacy and safety of landiolol.

Sinus tachycardia induced by high-dose catecholamine immediately after surgery requires a prompt strategy to control HR to avoid impaired impair cardiac systolic function. The present study provides important evidence supporting use of a safe and effective therapeutic regimen combining landiolol and inotropes.

## Limitations

The present study has several limitations. First, our sample size was small and only included 11 patients because concomitant use of catecholamine with β_1_-blocker agent is still not specified as a clinical treatment regimen. Second, our analysis was retrospective in nature. Prospective multicenter trials are required. However, a prospective design is not easy since a therapeutic approach should be dependent upon the judgment of the attending physician. Third, our comparison was between pre-administration and post-administration and involved only a single time point. We limited the study duration to only 1 h to minimize the effects of other clinical factors, such as fluid resuscitation, temperature recovery, and reduced bleeding. Fourth, this study did not have a control group; therefore, our findings are not definitive. Future studies are required to overcome these limitations.

## Conclusions

In the present study, low-dose β_1_-blocker landiolol therapy reduced HR and also increased SVI in patients who received sinus tachycardia-induced inotropic agents following on-pump cardiovascular surgery. While this study has some limitations in terms of low patient numbers and the study design, our findings suggest that landiolol is a promising therapy for the management of sinus tachycardia after CPB.

Reproducing these results using a large, appropriately powered and designed study could provide cardiovascular anesthesiologists and intensivists with a great addition to their pharmacological armamentarium.

## Data Availability

All data were retrieved from the institutional database and are available from the corresponding author upon reasonable request.

## Abbreviations

CAI: Cardiac agent index
CI: Cardiac index
CPB: Cardiopulmonary bypass
HR: Heart rate
SVI: Stroke volume index
